# Risk factors for subsidence in anterior cervical fusion with stand-alone polyetheretherketone (PEEK) cages: a review of 82 cases and 182 levels

**DOI:** 10.1007/s00402-014-2047-z

**Published:** 2014-08-07

**Authors:** Ting-Hsien Kao, Chen-Hao Wu, Yu-Ching Chou, Hsien-Te Chen, Wen-Hsien Chen, Hsi-Kai Tsou

**Affiliations:** 1 Functional Neurosurgery Division, Neurological Institute, Taichung Veterans General Hospital, Taichung, Taiwan, ROC; 2 Graduate Institute of Medical Science, National Defense Medical Center, Taipei, Taiwan, ROC; 3 Department of Acupressure Technology, Jen-Teh Junior College of Medicine, Nursing and Management, Miaoli County, Taiwan, ROC; 4 Department of Radiology, Taichung Veterans General Hospital, Taichung, Taiwan, ROC; 5 Department of Medical Imaging and Radiological Sciences, College of Heath Sciences, Central Taiwan University of Science and Technology, Taichung, Taiwan, ROC; 6 School of Public Health, National Defense Medical Center, Taipei, Taiwan, ROC; 7 School of Chinese Medicine, College of Chinese Medicine, China Medical University, Taichung, Taiwan, ROC; 8 Department of Orthopaedic Surgery, China Medical University Hospital, Taichung, Taiwan, ROC; 9 Department of Early Childhood Care and Education, Jen-Teh Junior College of Medicine, Nursing and Management, Miaoli County, Taiwan, ROC

**Keywords:** Anterior cervical discectomy, Fusion, Stand-alone, PEEK cage, Subsidence

## Abstract

**Introduction:**

To determine risk factors for subsidence in patients treated with anterior cervical discectomy and fusion (ACDF) and stand-alone polyetheretherketone (PEEK) cages.

**Materials and methods:**

Records of patients with degenerative spondylosis or traumatic disc herniation resulting in radiculopathy or myelopathy between C2 and C7 who underwent ACDF with stand-alone PEEK cages were retrospectively reviewed. Cages were filled with autogenous cancellous bone harvested from iliac crest or hydroxyapatite. Subsidence was defined as a decrease of 3 mm or more of anterior or posterior disc height from that measured on the postoperative radiograph. Eighty-two patients (32 males, 50 females; 182 treatment levels) were included in the analysis.

**Results:**

Most patients had 1–2 treatment levels (62.2 %), and 37.8 % had 3–4 treatment levels. Treatment levels were from C2–7. Of the 82 patients, cage subsidence occurred in 31 patients, and at 39 treatment levels. Multivariable analysis showed that subsidence was more likely to occur in patients with more than two treatment levels, and more likely to occur at treatment levels C5–7 than at levels C2–5. Subsidence was not associated with postoperative alignment change but associated with more disc height change (relatively oversized cage).

**Conclusion:**

Subsidence is associated with a greater number of treatment levels, treatment at C5–7 and relatively oversized cage use.

## Introduction

Cervical spondylosis-related disorders are common problems in modern countries [1]. If supportive medical treatment and physical therapy fail to relieve clinical symptoms, and neurological deficits due to bony spurs or disc herniation are present, surgery may be indicated. Anterior cervical discectomy and fusion (ACDF) have become the standard method of treatment, and ACDF can provide adequate neural decompression and good stabilization after arthrodesis is achieved [2–4].

Many materials are used to fuse adjacent vertebral bodies including autogenous bone graft, allograft, and artificial materials [2–9]. The purposes of these materials is to maintain disc height and alignment, widen the neuroforamen, and achieve good bony fusion. Interbody cages were developed by Dr. George Bagby decades ago, were first used in a horse with Wobbler’s syndrome, and bone ingrowth through the “Bagby basket,” and fusion between two vertebral bodies occurred. Since that time many advances have occurred, and interbody cages have become a primary method for ACDF, and although there are many cage designs and materials, most of them have been shown to provide an acceptable fusion rate [5, 6, 9]. Advantages of interbody cages include less donor site morbidity, shorter operation time and early postoperative ambulation. The use of stand-alone cages is common, and most cages are designed to resist pullout through an increased friction index or shape which keeps them more stable then iliac bone graft [10]. Anterior plating and screw fixation are commonly used to increase stability, prevent graft extrusion, and increase the bone fusion rate [11]. However, implant-related complications including screw pullout, plate and screw loosening, and dysphagia are a concern [11]. While placement of a stand-alone cage for single-level disease has been shown to be effective with good outcomes, their use in contiguous multi-level disease is still unclear [12, 13].

Subsidence is a concern with the use of stand-alone cages whether for single- or multi-level disease [13–15]. Some studies have shown there is a higher rate of subsidence with titanium vs. polyetheretherketone (PEEK) cages [16, 17]. Study has also shown that the rates of subsidence are similar with or without plate and screw fixation [18]. While subsidence is a known complication with the use of interbody cages for ACDF, whether or not subsidence affects long-term outcomes is unclear, and there are few studies examining subsidence with the use of stand-alone cages [12–18].

The purpose of this study was to determine the risk factors for subsidence in patients with cervical spondylosis-related disorders treated with ACDF and stand-alone PEEK cages.

## Methods

### Patients

In this study, the records of patients who were diagnosed with degenerative spondylosis or traumatic disc herniation resulting in radiculopathy or myelopathy between C2 and C7 and underwent ACDF with stand-alone PEEK cages from September 2005 to June 2009 were retrospectively reviewed and approved by the ethics committee at Taichung Veterans General Hospital (CE14062). The levels of treatment depended on the clinical presentation, physical examination findings, and imaging findings. All patients received preoperative flexion–extension dynamic cervical spine plain radiographs, and magnetic resonance imaging (MRI) studies. The intervertebral cages used were made of polyetheretherketone (PEEK) (Fidji^®^ Cervical Cage; Abbott/Zimmer, Warsaw, IN, USA). The material used to fill the cages was autogenous cancellous bone harvested from iliac crest, or hydroxyapatite, which has been shown to result in a similar fusion rate as when cancellous bone is used as the filling material [19].

### Surgical procedures

All patients were operated by the same experienced spinal surgeon, and a standardized right Smith-Robinson approach was used. Affected discs were totally excised, and bony spurs resulting in nerve or spinal cord compromise were removed. The adjacent cartilage was carefully shaved and removed, and care was taken to avoid excessive bony endplate destruction. The disc space was distracted and different sized trial cages were used until an appropriate sized cage was selected according to immobility of the trial cages following distractor removal. The cage was then filled with autogenous cancellous bone harvested from the anterior iliac crest or hydroxyapatite. The implant was inserted under fluoroscopic guidance to assure exact placement. The size and depth of the implant was checked by the fluoroscopy immediately after placement at each level. After completing each level, the wound was cleaned and closed in a standard manner.

### Radiographic assessment

**Fig. 1:**
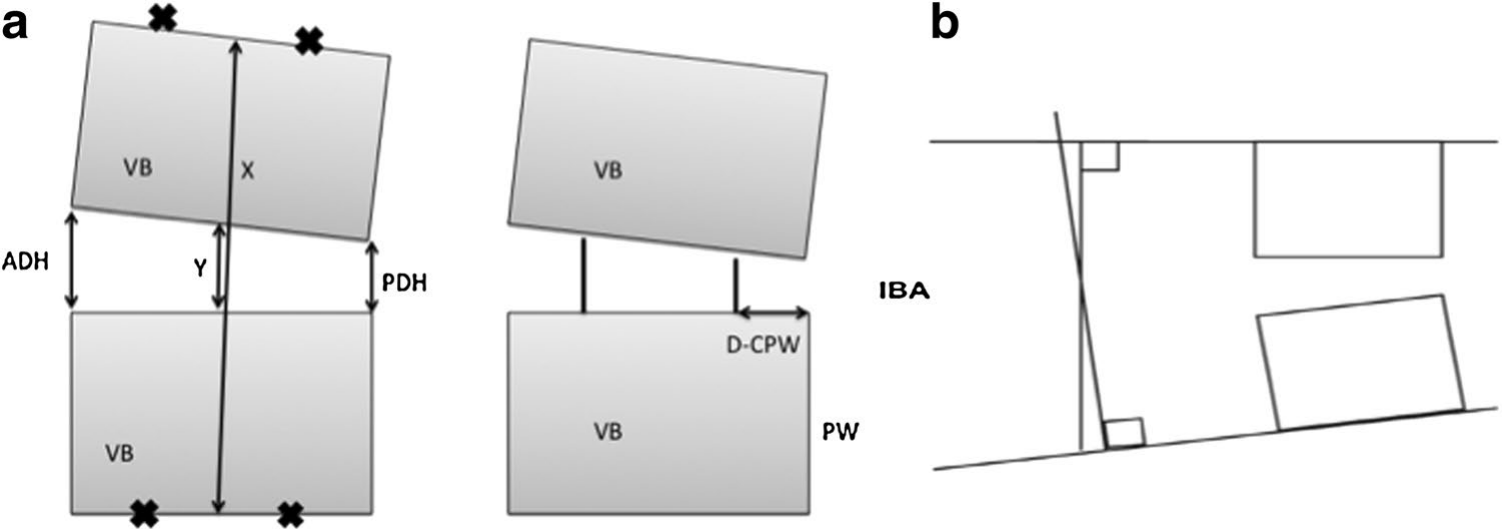
Measurement used in the analysis. *ADH* anterior disc height, *PDH* posterior disc height, *x* distance between the midpoints of the upper and lower endplates, *y* distance between the midpoints of the lower and upper endplates, *VB* vertebral body, *D-CPW* distance between the posterior titanium line of within the PEEK cage and posterior wall of the vertebral body, *IBA* interbody angle

All patients received postoperative anteroposterior (AP) and lateral plain radiographs within 1 week after surgery. Patients were regularly followed-up, and plain radiographs of the cervical spine were obtained at 1, 3, 6, and 12 months after surgery. All radiographs were assessed by two experienced neuroradiologists blinded to the clinical status of the patients. Subsidence, fusion, and migration of the PEEK cages were evaluated on the basis of the lateral radiographs. Spinal fusion was defined as the presence of bony trabeculae across the graft-host interfaces, trabeculae bridging bone formation at the anterior and/or posterior cortex of the involved vertebral bodies, and a hazy interface between the cage and the vertebral endplate. Absence of such bridges or the presence of an anteroposterior discontinuation was classified as non-fusion. Subsidence was defined according to a method previously described [13]. Briefly, subsidence was considered to have occurred if either the anterior disc height (ADH) or posterior disc height (PDH) decreased more than 3 mm from that measured on the postoperative radiograph. The ADH, PDH, distance between the posterior margin of the titanium line of the cage (a radiopaque marker within the PEEK cage) and the posterior wall of the vertebral body (D-CPW), and interbody angle (IBA) were calculated as previously described [5] (Fig. 1). The interbody disc height ratio (IDHR) was defined differently with the previous article and defined as adjacent body height (*x*)/disc height ratio (*y*) (Fig. 1). If the IDHR value increased after operation, the cage size is relatively under-estimated and vice versa. Difference between preoperative and postoperative disc height was defined as the ratio of preoperative IDHR/postoperative IDHR and it means relatively oversized interbody cage will cause the value increased and vice versa. In this study, the change in the ADH and PDH ratio was used as an indication of the correction of alignment after surgery.

### Statistical analysis

Data were expressed as mean ± standard deviation (SD) for continuous variables, and number (percentage) for categorical variables. Characteristics between the groups with subsidence of cage and that without subsidence (yes or no) were compared by the two independent samples *t* test for continuous variables, and the Fisher’s exact test for categorical variables. The non-parametric Mann–Whitney test was performed to compare the number of treatment levels between male and female. A multiple generalized linear model with generalized estimating equations (GEEs) was performed with two steps to estimate odds ratios (ORs) with 95 % confidence intervals (CIs) of subsidence risk for the potential risk factors. First, variables with a *p* value <0.1 in univariable analysis were identified, and second, these variables were included in a stepwise manner in the multivariable analysis by the forward conditional method. The Statistical Package for Social Sciences version 19.0 (SPSS, Inc., Chicago, IL, USA) was used for all statistical analyses. Values of *p* < 0.05 were considered to indicate statistically significance.

## Results

A total of 82 patients, 32 male and 50 female, were included in the analysis. Most of the patients were diagnosed with degenerative radiculopathy or myeloradiculopathy (*n* = 77), and five were diagnosed as post-traumatic myeloradiculopathy without other associated injuries. Most of the patients had 1–2 treatment levels (62.2 %), and 37.8 % had 3–4 treatment levels. In total, 182 treatment levels of the 82 patients were analyzed. Treatment levels were from C2 to C7. Of the 82 patients, cage subsidence occurred in 31 patients during postoperative follow up (Table 1). Cage subsidence was not significantly associated with age (*p* = 0.231), but significantly associated with gender; 54.8 % of the 31 patients with subsidence were male, but only 29.4 % of the 51 patients without subsidence were male (*p* = 0.035). The number of discectomies was significantly associated with subsidence of the cage; 58.1 % of patients with subsidence group had 3–4 treatment levels, but only 25.5 % of patients without subsidence had 3–4 treatment levels (*p* = 0.005) (Table 1).

**Table 1:** Analysis of subsidence by patient characteristics

	Total (*n* = 82)	Subsidence of cage		*p* value
		Yes (*n* = 31)	No (*n* = 51)	
Age (year)	57.1 ± 12.7	59.2 ± 13.3	55.7 ± 12.3	0.231
Gender				0.035
Male	32 (39.0)	17 (54.8)	15 (29.4)	
Female	50 (61.0)	14 (45.2)	36 (70.6)	
Diagnosis				0.065
CSR and CSMR	77 (93.9)	27 (87.1)	50 (98.0)	
Trauma^a^	5 (6.1)	4 (12.9)	1 (2.0)	
Number of discectomies				0.002
1	16 (19.5)	1 (3.2)	15 (29.4)	
2	35 (42.7)	12 (38.7)	23 (45.1)	
3	28 (34.1)	16 (51.6)	12 (23.5)	
4	3 (3.7)	2 (6.5)	1 (2.0)	
Number of discectomies				0.005
≤2	51 (62.2)	13 (41.9)	38 (74.5)	
>2	31 (37.8)	18 (58.1)	13 (25.5)	

Data are presented as number (percentage), except for age which is presented as mean ± standard deviation

*CSR* cervical spondylotic radiculopathy, *CSMR* cervical spondylotic myeloradiculopathy

^a^Traffic accident or fall without endplate destruction on imaging studies

The analysis of subsidence by treatment level is presented in Table 2. Subsidence was more common when the treatment levels were C5–6 and C6–7 than when the treatment levels were C2–3, C3–4, and C4–5 (*p* = 0.003). The more disc height change (relatively oversized cage) was significantly higher in the subsidence group than in the non-subsidence group (0.677 vs. 0.747, *p* = 0.024). Subsidence was not significantly associated with alignment change (*p* = 0.352).

**Table 2:** Analysis of subsidence by treatment level

	Total (*n* = 182)	Subsidence of cage		*p* value
		Yes (*n* = 39)	No (*n* = 143)	
Treatment level				0.003*
C2–3, C3–4, C4–5	100 (54.9)	13 (33.3)	87 (60.8)	
C5–6, C6–7	82 (45.1)	26 (66.7)	56 (39.2)	
Disc height change^a^	0.732 ± 0.172	0.677 ± 0.146	0.747 ± 0.176	0.024*
Alignment change^b^	1.181 ± 0.566	1.282 ± 0.810	1.154 ± 0.479	0.352

Data are presented as number (percentage) or mean ± standard deviation

^a^Disc height change defined as the difference of the ratio of vertebra height and disc height between preoperative and postoperative radiographs

^b^Alignment change defined as the difference of the ratio of anterior disc height (ADH) and posterior disc height (PDH) between preoperative and postoperative radiographs

* Significant impact on the occurrence of subsidence

Thirty-nine levels (21.4 %) in 31 patients (37.8 %) were found as cage subsidence radiologically. The subsidence rates of all treatment levels increased during the 12 months after surgery, and were 0 % at 1 week, 2.2 % at 1 month, 6.6 % at 3 months, 10.4 % at 6 months, and to 21.4 % at 12 months. There were no perioperative major complications or subsidence-related symptoms that required treatment during the 12 months follow-up postoperatively.

Results of the univariable and multivariable analysis of risk factors associated with subsidence are shown in Table 3. In univariable analyses, patients with trauma were more likely to experience subsidence than those diagnosed with cervical spondylotic radiculopathy (CSR) or cervical spondylotic myeloradiculopathy (CSMR) (OR = 1.83, *p* = 0.025), and male were more likely to experience subsidence than female (OR = 2.43, *p* = 0.025). Subsidence was more likely to occur at treatment levels C5–7 than C2–5 (OR = 3.96, *p* < 0.001). In addition, an increased disc height change was associated with a lower risk of subsidence (OR = 0.80, *p* = 0.018). In other words, relatively oversized cage use (decreased IDHR postoperatively) was associated with a higher risk of subsidence. Variables with *p* value <0.1 in univariable analysis were stepwise included in the multivariable analysis by the forward conditional method.

**Table 3:** Evaluation of risk factors of subsidence for the 182 treatment levels

	Univariable analysis		Multivariable analysis	
	OR (95 % CI)	*p* value	OR (95 % CI)	*p* value
Age (year)	1.02 (0.99, 1.06)	0.247		
Gender				
Male	2.43 (1.12, 5.27)	0.025*		
Female	Reference group			
Diagnosis				
Trauma	1.83 (1.08, 3.11)	0.025*		
CSR or CSMR	Reference group			
Number of treatment levels				
>2	2.08 (0.96, 4.50)	0.062	2.47 (1.05, 5.78)	0.038*
≤2	Reference group		Reference group	
Treatment levels				
C5–7	3.96 (1.84, 8.49)	<0.001*	3.48 (1.73, 6.99)	<0.001*
C2–5	Reference group		Reference group	
Disc height change^a^	0.80 (0.671, 0.963)	0.018*	0.84 (0.69, 1.02)	0.078
Alignment change^b^	1.45 (0.86, 2.45)	0.164		

Variables with a *p* value <0.1 in univariable analysis were stepwise included in the multivariable analysis by the forward conditional method

*CSR* cervical spondylotic radiculopathy, *CSMR* cervical spondylotic myeloradiculopathy

* Significant impact on the occurrence of subsidence

^a^Disc height change defined as the difference of the ratio of vertebra height and disc height between preoperative and postoperative radiographs

^b^Alignment change defined as the difference of the ratio of anterior disc height (ADH) and posterior disc height (PDH) between preoperative and postoperative radiographs

Univariable analysis indicated that male had significantly more treatment levels than female (median with range: 3 [1–4] vs. 2 [1–3], *p* = 0.034), so it appeared that gender was a confounder for the association of treatment level number and subsidence occurrence. Treatment level number, not gender, was found to be significantly associated with subsidence in the multivariable analysis; patients with more than two treatment levels were more likely to experience subsidence than those with 1–2 treatment levels (OR = 2.47, *p* = 0.038). In addition, subsidence was more likely to occur at treatment levels C5–7 than at levels C2–5 (OR = 3.48, *p* < 0.001).

**Fig. 2:**
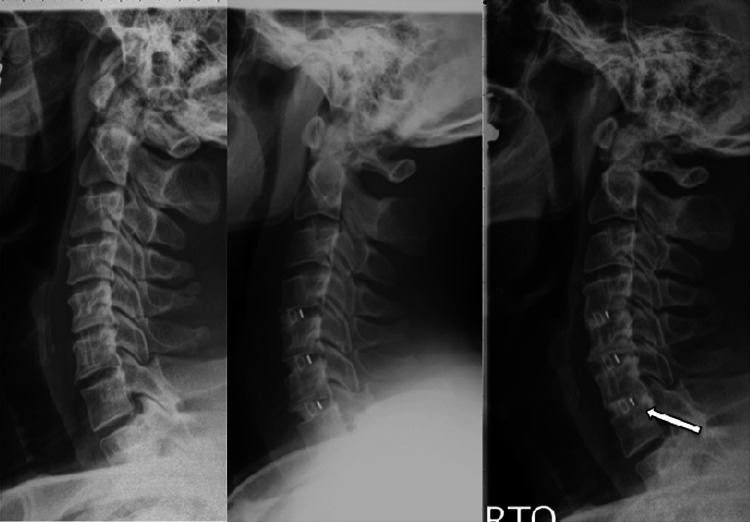
Example of fusion through the interbody space in a patient who underwent a three level discectomy. *Left* to *right* preoperative, postoperative 1 and 6 months

**Fig. 3:**
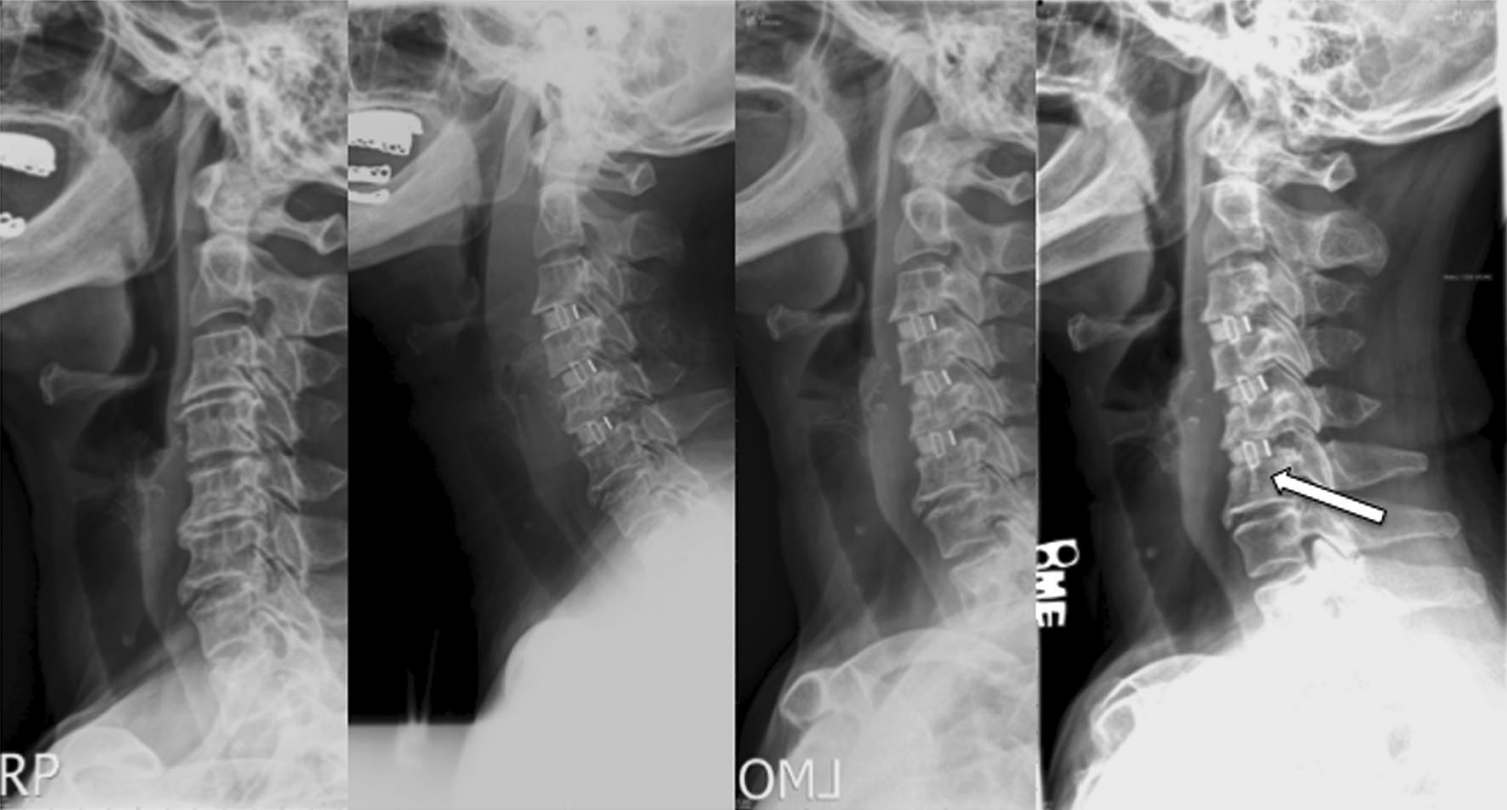
Example of interbody cage subsidence in a patient who underwent a three level discectomy. *Left* to *right* preoperative, postoperative 1, 6, 12 months

Representative radiographs illustrating fusion and subsidence through the interbody space are shown in Figs. 2 and 3, respectively.

## Discussion

This study examined the risk factors of subsidence in patients with cervical spondylosis-related disorders treated with ACDF and stand-alone PEEK cages. The results showed that subsidence was more likely to occur in patients with more than two treatment levels, and more likely to occur at treatment levels C5–7 than at levels C2–5. Subsidence was associated with more disc height change (relatively oversized cage) but not postoperative alignment change.

Anterior cervical discectomy was introduced by Dr. Cloward, and has become the gold standard for the treatment for cervical disc-related disease [20]. After decompression, placing an interbody bone graft can preserve disc height, widen the neuroforamen, and normalize cervical spine alignment. Rigid arthrodesis must be achieved after ACDF because of the possibility of graft dislodgement or pseudoarthrosis-related complications [21]. In the past years, autogenous bone graft and allograft with plating have been used with good fusion rates [2–9]. However, donor site morbidity can occur with autogenous iliac bone graft harvesting, and allograft is not available at all hospitals. The interbody cage was developed in 1979, and a titanium cervical cage for spinal fusion surgery in humans was approved by the United States Food and Drug Administration (US FDA) in 1996. Advantages of cervical interbody cages include shorter operation time, no donor site morbidity associated with bone harvesting, and a lower possibility of graft collapse [22, 23].

Interbody cages are designed with a high friction index to prevent pullout and increase the fusion rate, and stand-alone cervical cages are used for single- and multi-level interbody fusion by many spinal surgeons [5, 12, 13, 24–27]. Study has shown that while fusion with interbody cages may be delayed as compared to traditional bone graft, the fusion rates are similar as are the overall outcomes [6, 9, 28, 29]. Since the consistency of titanium material is not the same as the cortical bone adjacent to the cage, other materials have been developed to decrease the transition force between the cage and the vertebral bodies, for example PEEK, carbon fiber and trabecular metal cages [2, 5–7, 9, 30].

Subsidence is defined as the sinking of an object with a greater elasticity modulus (e.g., cage or spacer) into an object with a lower elasticity modulus (e.g., vertebral body) [31]. In cases of ADCF with an interbody cage, this sinking can result in changes to the spinal geometry. The amount of subsidence is directly proportional to the load pressure and the difference in the elastic modulus between the two materials, and inversely proportional to the area of the interface between the two objects (i.e., cage and vertebral endplate) [31]. A large amount of subsidence can, in addition to altering spinal geometry, cause breakage or pullout of screws. Zhang et al. [32] have suggested that subsidence may be due to a process of bone incorporation between cages and endplates, and that further study is necessary to determine the clinical significance of subsidence. Similarly, Borm et al. [12] have suggested that subsidence may be the normal process of bone resorption and remodeling until fusion is established. Truumees et al. [33] suggested that subsidence may be caused by over-distraction itself, rather than absence of a fixation device.

Chen et al. [17] reported that cage subsidence was more common with titanium cages than PEEK cages when used for ACDF in patients with three-level cervical spondylotic myelopathy (34.5 vs. 5.4 %, respectively); however, the occurrence of subsidence did not affect the fusion rates which were similar between the two groups. Similarly, Cabraja et al. [16] treated 154 patients with degenerative cervical disc disease with ACDF and found a subsidence rate of 20.5 % with titanium cages and 14.3 % with PEEK cages, and solid arthrodesis occurred in 93 % of the patients with titanium cages and 88 % of those with PEEK cages. The higher rate of subsidence with titanium cages is not unexpected as the elasticity modulus of titanium is greater than that of PEEK [31]. Wang et al. [34] treated 16 patients with two level non-contiguous cervical degenerative disc disease with ACDF and stand-alone PEEK cages without plating and reported that three cages in two patients subsided, but this did not affect the fusion rate or final outcomes. Wu et al. [35] treated 57 consecutive patients (68 levels) with stand-alone titanium cages for degenerative cervical disc disease, and found that cage subsidence had occurred in 13 cages (19.1 %) at the 3 month follow-up, but there was no relationship between cage subsidence and fusion. They also found there was no difference in the recovery rate of Japanese Orthopedic Association (JOA) cervical myelopathy score or difference in neck and radicular pain between the subsidence and non-subsidence groups. The findings led the authors to conclude that cage subsidence does not have a significant impact on long-term clinical outcomes. A recent systematic review of the literature by Karikari et al. [15] also concluded that subsidence irrespective of the measurement technique or definition does not seem to have a significant impact on successful fusion or clinical outcome in patients undergoing ACDF.

Subsidence is a concern with the use of interbody cages because it causes disc height narrowing, which can decrease the neuroforamen space created by cage distraction [13]. In the current study, the cage size was chosen by surgeon experience of the distraction force required or alignment after using trial and error method. However, there are no objective parameters to use for determining the correct size cage or for predicting the clinical outcome. Factors that have been associated with an increased risk of subsidence include poor surgical technique such as over distraction, and wrong cage size [5, 13, 27, 36]. Endplate preparation may be a factor in the occurrence of subsidence [27, 36]. Study has shown that early consolidation occurs if the endplate is removed, whereas without the removal of the endplate a fusion rate of 80 % can be achieved after 6 months [37]. The bone formation processes may be different when the endplate is removed as compared to when the endplate is intact; the former may result in spongy bone growing through the cage and the later may result in chondrogenic new bone formation [38]. It has also been reported that the incidence of subsidence is greater at the C6–7 levels [39]. Subsidence is also associated with the severity of osteoporosis [40].

There are some limitations of this study. First, while the accuracy of linear measurements on a picture archiving and communication system (PACS) is acceptable for computed tomography (CT) scans, it is inadequate for digital plain radiographs without the use of an internal calibration [41]. For this reason we used the ratio rather than the actual disc height to achieve a more precise analysis. However, cage subsidence was still defined as sinking of 3 mm or more into the endplate plane. In addition, the posterior longitudinal ligament (PLL) was excised in some cases depending on the intraoperative findings. For example, if the nerve or spinal cord was compressed by local or complete PLL ossification it was excised. On the contrary, the PLL was not completely excised if compression was due to simple nucleus pulposus herniation. Excision of the PLL was not a variable included in the analysis, and stability has been shown to be influenced by the PLL [15]. Lastly, the number of patients and the number of individual levels that received surgery were relatively small.

In conclusion, subsidence was more likely to occur in patients with more than two treatment levels, and more likely to occur at treatment levels C5–7 than at levels C2–5. Subsidence was not associated with postoperative alignment change but associated with more disc height change (relatively oversized cage). While the occurrence of subsidence does not appear to affect fusion or long-term clinical outcomes, further study is required to evaluate the significance of subsidence occurring after ACDF with PEEK cages.
